# Late preterm: reorganization of Neonatology Unit

**DOI:** 10.1186/1824-7288-40-S2-A49

**Published:** 2014-10-09

**Authors:** Anna Maria Alessi, Luciana Leva, Elisabetta Villa, Mario Barbarini

**Affiliations:** 1 Neonatology and Neonatal Intensive Care Unit, Azienda Ospedaliera Ospedale Sant’Anna, Como, Italy

## 

Our reorganization project arises from the need to assist in Neonatology newborns with GA (Gestational Age) ≥ 34 weeks and/or birth weight ≥ 1800 g in good cardiorespiratory conditions and newborns still partially fed by nasogatric tube.

Up to June 2010 our Neonatology usually assisted late-preterm newborns but our new needs require to increase patients' complexity, maintaning pre-existent Staff.

In accordance with the informations provided by the legislation in force about equipment and staff requirement, we made an organization change that led to the transition from nursing care organized by tasks to a newborn assistance organized by complexity of care.

This change leads to the identification of a nurse which is the person in charge of the baby, ensures continuity of care and promotes improvement of personalization of the assistance.

We also carried out assistance modality which guarantees the respect of the skills of the individual infant in relation to his neuro-evolutionary development, resulting in a greater involvement of parents in the process of care.

We also made a review of nursing documentation because the pre-existent one was not suitable to record adequately the needs and observations necessary for this type of patients: we introduced a “Survey of parameters” for every newborn, defined by a specific protocol, which needs a particular observation.

After an initial trial of this new tool in Neonatology, we decided to extend its application even in the delivery room involving midwives and this change ensures greater safety of the newborn and a continuing process of documentation, safeguarding a good start bounding.

This experience of sharing a course so far kept strictly separate led to a marked improvement over the documents relating to the early hours of the infant's life and promoted greater collaboration between the different disciplines involved in the care process.

